# Intracerebral opportunistic infections caused by immunosuppressants after orthotopic liver transplantation: Report of two cases and literature review

**DOI:** 10.3389/fimmu.2022.1003254

**Published:** 2022-12-05

**Authors:** Yafei Guo, Zebin Zhu, Wei Cai, Shengwei Tao, Dalong Yin

**Affiliations:** Department of Hepatobiliary Surgery and Organ Transplantation Center, the First Affiliated Hospital of University of Science and Technology of China, He Fei, Anhui, China

**Keywords:** immunosuppressant, opportunistic infections, orthotopic liver transplantation, intracranial infections, HHV-6 encephalitis

## Abstract

Central nervous system (CNS) infections in adults are rare because of normal immunity and the existence of the blood brain barrier, which prevents the invasion of pathogenic microorganisms. Liver transplant recipients are at an increased risk of opportunistic infections (OI) due to immunosuppressive therapy compared to those with normal immunity. Early diagnosis and timely implementation of treatment are critical for the successful treatment of these infections. We present two cases of intracerebral OI after orthotopic liver transplantation (OLT), with different clinical presentations. Patient 1 presented with epileptic seizures, mainly manifested as unresponsiveness, unconsciousness, and coma complicated with involuntary limb twitching. Patient 2 presented with a consciousness disorder, mainly manifested as unclear consciousness content, poor orientation, calculation power, and logical ability. Next-generation sequencing (NGS) examination of the cerebrospinal fluid confirmed human herpesvirus 6 B (HHV-6B) infection in patient 1 and intracranial Aspergillus infection in patient 2. Intracranial OI has insidious onset and atypical clinical manifestations. NGS can allow for the proper diagnosis and monitoring of the effects of treatment.

## Introduction

Liver transplantation is the gold standard treatment for end-stage liver disease. With the development of immunosuppressive agents, the incidence of rejection has significantly reduced; however, the risk of opportunistic infections (OI) after liver transplantation is gradually increasing, which mostly occur within the intermediate period (1–6 months after liver transplantation) owing to persistent intensive immunosuppression (1). The most common pathogens causing opportunistic central nervous system (CNS) infections are fungi and viruses, whereas bacteria and protozoans are uncommon. Owing to improvements in immunosuppressive therapy and routine surveillance, the prevalence of these infections has decreased from 7% to 1–2% (2, 3). Herpes viruses (herpes simplex virus HSV1/2, human herpesvirus HHV-6, and HHV-8), Epstein-Barr virus (EBV), cytomegalovirus (CMV), Cryptococcus, and Aspergillus are the most common pathogens (4). Clinical manifestations include fever, headache, meningitis, Kernig and Brudzinski signs, new-onset seizures, papilloedema, altered sensorium, and/or focal neurological deficits (5). These manifestations may be subtle or absent because of the use of immunosuppressive agents. Virus and fungi OI continue to be a significant cause of morbidity and mortality in patients with CNS involvement, owing to the poor CNS penetration of antifungal and antiviral medications. Herein, we report the successful diagnosis and treatment of two cases of CNS OI that occurred within the first month after orthotopic liver transplantation (OLT). The diagnosis and treatment of the two cases are summarized in **Table 1**.

**Table 1:** Two cases of CNS opportunistic infections.

Liver transplantation	Patient 1 (P1)	Patient 2 (P2)
**Recipient demographics**		
** Age (years)**	56 years old	44 years old
** Gender**	Male	Male
** Blood type**	Blood type B	Blood type AB
** Primary disease**	HBV-associated liver cirrhosis	HBV-associated ACLF
** MELD score**	20	29
** Child-Pugh score**	9	13
** BMI-kg/m^2^**	20.2	27.7
** ICU stay post-transplantation**	3 days	14 days
**Donor demographics**		
**Age**	29 years old	54 years old
**Gender**	Male	Male
**Blood type**	Blood type B	Blood type AB
**Primary disease**	Craniocerebral trauma	Cerebral hemorrhage
**Donor type**	DBD	DCD
**ICU stay before procurement**	1 day	1 day
**Donor liver steatosis**	Mild steatosis (10%)	Normal
**Virology and fungal check**	Negative	Negative
**Immunosuppressive regimen**		
**During transplantation **	Methylprednisolone + Basiliximab	Methylprednisolone + Basiliximab
**After transplantation**	Tacrolimus + Mycophenolate mofetil + Corticosteroid; Basiliximab 20mg on POD 4	Tacrolimus + Mycophenolate mofetil + Corticosteroid; Basiliximab 20mg on POD4
**During opportunistic infection**	Corticosteroid 100mg/day alone	Tacrolimus+Sirolimus
**Intracerebral infection**		
**Organism**	HHV-6B	Aspergillus
**Infection time**	On POD 18	On POD 7
**Clinical presentation**	Encephalitis	Brain abscess
**Diagnosis**	NGS of CSF	NGS of CSF
**Treatment**	Ganciclovir	Voriconazole + Fluconazole

ACLF, Acute-on-chronic liver failure (ACLF); BMI, Body mass index (BMI); CNS, Central nervous system (CNS); CSF, Cerebrospinal fluid (CSF); DBD, Donation after brain death (DBD); DCD, Donation after circulatory death (DCD); HBV, Hepatitis B virus (HBV); HHV-6B, Human herpesvirus 6 B (HHV-6B); ICU, Intensive care unit (ICU); NGS, Next-generation sequencing (NGS);POD, Postoperative day (POD); MELD, Model for end-stage liver disease (MELD).

## Case reports

**Figure 1:**
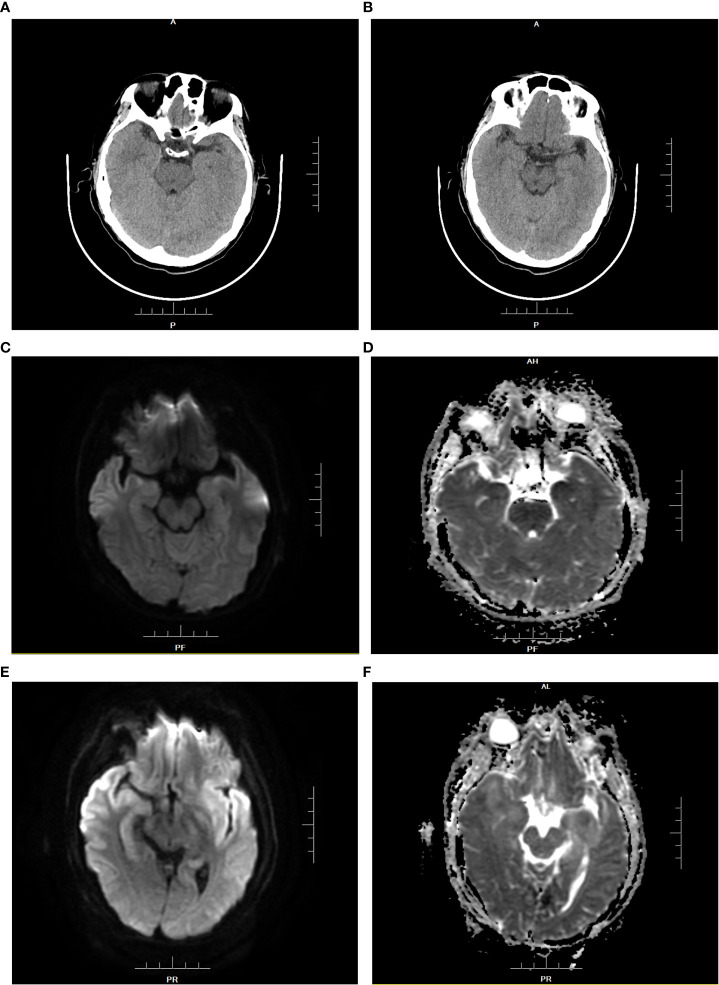
Brain CT and MRI of patient 1 before medication **(A–D)**. **(A, B)**: The density of the inferior pole of both the temporal lobes was slightly decreased. **(C)**: DWI showed that white matter signals under the gray matter of the inferior temporal pole were limited on both sides. **(D)**: ADC showed symmetrical low signal changes.Brain MRI findings of patient 1 at the diagnosis of HHV-6B encephalitis **(E, F)**. **(E)**: DWI showed that bilateral inferior temporal poles were diffusively limited and symmetrical, with upwards involvement of the bilateral basal ganglia and subgray matter region of the medial lateral temporal lobe. **(F)**: ADC presented high signal changes.

Patient 1 (P1) was a 56-year-old man who underwent OLT for HBV-associated liver cirrhosis and hepatocellular carcinoma. The initial recovery from transplantation was excellent, without early operation-related complications. Methylprednisolone 1.0 g and basiliximab (20 mg) were administered during the anhepatic phase, and basiliximab (20 mg) was administered again on postoperative day 4 (POD 4). Baseline immunosuppression after OLT included tacrolimus (Tac, 3 mg bid, C0 3-8 ng/ml), mycophenolate mofetil (MMF, 500 mg bid), and corticosteroids (100 mg tapered to 5 mg on POD 8). Considering that the patient had hepatocellular carcinoma, baseline immunosuppression was adjusted to tacrolimus plus sirolimus, while MMF and corticosteroids were discontinued on POD 14. On POD 18, the patient suddenly appeared delirious, unconscious, and unresponsive, accompanied by involuntary twitching of the limbs. There were marked changes in vital signs: blood pressure was 170/100 mmHg,; heart rate, 150 beats per minute; oxygen saturation 90–96%, temperature 36.6°C, and respiratory rate 22–25 breaths per minute. Physical examination of the cranial nerves and neurological examination of the upper and lower limbs revealed no abnormalities. Magnetic resonance imaging (MRI) and computed tomography (CT) of the brain revealed slight ischemic changes, without other obvious abnormalities (**Figures 1A–D**). Electroencephalogram examination revealed seizures. Routine and biochemical tests of CSF did not reveal any obvious abnormalities. CSF cultures for bacteria, fungi, and other viruses and peripheral blood examination for cytomegalovirus were negative. CSF pathogen microbial NGS confirmed the presence of 30691 human herpesvirus 6 B (HHV-6B) DNA sequences. Real-time fluorescent quantitative polymerase chain reaction (RT-qPCR) detected 12000 copies/mL HHV-6B DNA in the CSF. Antiviral therapy with ganciclovir (250 mg intravenously every 12 h) was administered. The immunosuppressive regimen was changed to corticosteroid maintenance alone at a dosage of 100 mg/day, and tacrolimus and sirolimus were discontinued. After 4 days of treatment, the seizure symptoms disappeared, and the confusion, headache, and involuntary movements improved. Re-examination of the brain MRI suggested intracranial infectious lesions (**Figures 1E, F**). Re-examination of CSF revealed 29 HHV-6B DNA sequences two weeks later, which was significantly less than before (30691 HHV-6B DNA sequences). The CSF for HHV-6B was negative, and the neurological symptoms returned to normal after 4 weeks of treatment. Ganciclovir was reduced to 150 mg once daily maintenance therapy, with gradual withdrawal of corticosteroids and resumption of basic immunosuppression (tacrolimus combined with sirolimus).

**Figure 2:**
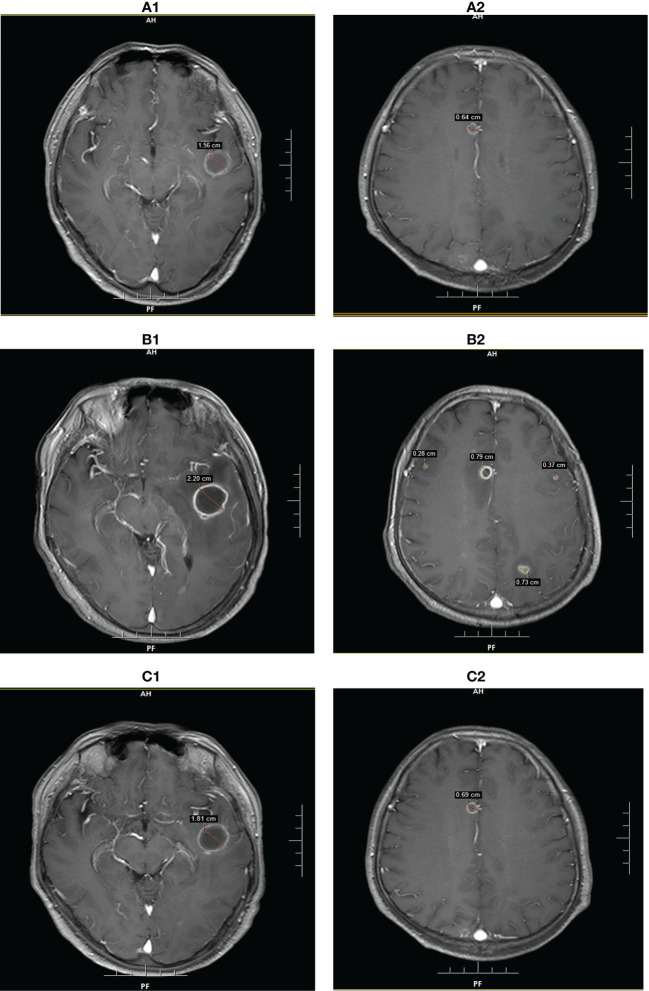
Brain MRI findings of patient 2 at the diagnosis of intracranial Aspergillus infection. **(A)**: MRI showed two lesions with diameters of 1.56 cm and 0.64 cm on POD 7. **(B)**: MRI showed five lesions with diameters of 2.20 cm, 0.79 cm, 0.73 cm, 0.37 cm, and 0.28 cm on POD 28. **(C)**: MRI showed two lesions with diameters of 1.81 cm and 0.69 cm on POD 65.

Patient 2 (P2) was a 44-year-old man who underwent OLT for acute-on-chronic liver failure (ACLF). The patient had chronic hepatitis B for several years and received irregular treatment. During the transplant operation, the patient suffered severe post-reperfusion syndrome (PRS) due to hyperkalemia, that is, cardiac arrest after reperfusion. After cardiopulmonary resuscitation, the heartbeat and circulation returned to normal levels. Nevertheless, the initial recovery from transplantation was excellent, and there were no early operation-related complications. Intraoperative immune induction and postoperative baseline immunosuppression were the same as those in Patient 1. One week after transplantation, the patient exhibited a consciousness disorder, mainly manifested as unclear content of consciousness, loss of orientation, calculation ability, and logical ability. The vital signs were stable, except for a slight fever, with a maximum temperature of 38.0°C. Brain magnetic resonance imaging (MRI) revealed two abnormal signals in the left temporal lobe and right frontal lobe, and the possibility of infection was considered (**Figure 2A**). CSF routine, biochemistry, and culture also showed no abnormalities, but NGS confirmed an invasive Aspergillus infection in the CSF. Based on these guidelines, voriconazole was selected as the antifungal therapy. Meanwhile, the dosage of tacrolimus was adjusted to maintain the concentration below 8 ng/ml. After three weeks of antifungal treatment, the patient’s neurological symptoms slightly improved, but re-examination of brain MRI showed that the lesion was larger than before (**Figure 2B**). A second NGS of the CSF showed Candida, but no Aspergillus. After multidisciplinary consultation and discussion, a biopsy was performed to confirm the pathology; however, the patient and his family refused to do so. Considering the antifungal effect of sirolimus, immunosuppression was adjusted to sirolimus monotherapy for maintenance therapy. Meanwhile, fluconazole was added for antifungal therapy and the plasma concentration was maintained between 4.5 mg/mL and 5.5 mg/mL. After 2 weeks of combined treatment, re-examination of brain MRI showed that the lesions did not increase significantly, and the neurological symptoms were significantly improved. During the following three weeks, antifungal therapy was maintained, and re-examination of the brain MRI showed that the infected lesions were smaller than before (**Figure 2C**). Immunosuppression was gradually restored by tacrolimus combined with sirolimus. After comprehensive treatment, the patient recovered well and was discharged three months after liver transplantation.

## Discussion

The risk of opportunistic infections in the central nervous system (CNS) opportunistic infections (OI), which often presents as a diagnostic challenge, is related to the nature and intensity of immunosuppression and the infectious exposures of the organ recipient and donor (6). The pathogens associated with these infections are often found to have low virulence in immunocompetent hosts and include various bacteria, parasites, fungi, or viruses (7). These infections can present as various clinical syndromes, including meningitis, encephalitis, space-occupying lesions, stroke-like presentations, or even neoplastic manifestations. CSF evaluation is required for definitive diagnosis of CNS OI. Some common clinical organisms responsible for CNS OI are listed in **Table 2**.

**Table 2:** List of common CNS opportunistic infections.

Type	Organism	Clinical Presentation	Treatment
**Bacterial**	Listeria monocytogenes Nocardia asteroides Mycobacterium tuberculosis	Rhomboencephalitis, meningitis Meningitis, cerebritis, mass lesion Basilar meningitis, tuberculoma	Ampicillin, gentamicin Trimethoprim-sulfamethaxole Rifampin+isoniazid+pyrazinamide+ethambutol+pyridoxine
**Fungi**	Cryptococcus Aspergillus Candida Blastomyces Histoplasma Coccidiomycosis Mucormycosis	Meningitis, mass lesion Brain abscess, hemorrhage Meningitis, abscess Brain abscess Meningitis Basilar meningitis Rhinocerebral infection	Amphotericin Voriconazole Amphotericin Amphotericin + itraconazole Amphotericin Amphotericin Amphotericin
**Viruses**	CMV EBV HSV-1/HSV-2 HHV-6 JC virus (JCV) Varicella zoster virus (VZV)	Encephalitis Encephalitis Encephalitis Limbic encephalitis PML Shingles, GuillainBarre syndrome	Ganciclovir, foscarnet, CMV Immunoglobulin Acyclovir, ganciclovir Acyclovir Ganciclovir, foscarnet No medical therapy Acyclovir, VZV immunoglobulin
**Parasites**	Toxoplasmosis	Space-occupying lesion	Pyrimethamine+sulfadiazine+folinic acid

CMV, cytomegalovirus (CMV); EBV, Epstein‒Barr virus (EBV); HSV, herpes simplex virus (HSV); HHV-6, human herpesvirus 6 (HHV-6); PML, Progressive multi-focal leukoencephalopathy (PML), CNS, Central nervous system (CNS)

Human herpesvirus 6 (HHV-6) is a common collective name for two distinct viruses, human herpesvirus 6A (HHV-6A) and human herpesvirus 6B (HHV-6B) (8). HHV-6 belongs to the beta-herpesvirus subfamily, which is widespread, with a seroprevalence of > 90% in adults (9). In liver transplant recipients, infections caused by this virus are almost always HHV-6B (10). HHV-6B efficiently infects CD4^+^ T cells and acts as a receptor for CD134 (OX40), a member of the TNF superfamily that antagonizes regulatory T-cell (Treg) activity (11, 12). Primary infection is usually associated with a benign skin rash in infants (exanthema subitum), but reactivation from latency can lead to severe diseases such as hepatic, neurological, and disseminated infections, especially in immunocompromised patients (13). HHV-6B reactivation has been associated with aberrant immune reconstitution and acute graft-versus-host disease (aGVHD) after hematopoietic cell transplantation (HCT) (14). HHV-6B reactivation after liver transplantation is mostly asymptomatic but can be associated with fever, hepatitis, and encephalitis (15). A retrospective study showed a correlation between HHV-6B infection and symptoms of encephalitis in seven (35%) of 20 liver transplant recipients (16). The virus mainly affects the limbic system of the brain, including the hippocampus, causing limbic encephalitis. Clinical symptoms can be characterized by headache, mental disorders, memory loss, confusion, ataxia, convulsions, seizures, typically characterized with or without seizures of confusion and amnesia, and gradual progress to confusion and coma (7, 17, 18). Hyponatremia due to syndrome of inappropriate antidiuretic hormone (SIADH) has been noted in HHV-6B encephalitis (7). Typical MRI shows hyperintensities within the uncus, amygdala, entorhinal area, and hippocampus on T2, fluid-attenuated inversion recovery (FLAIR), and diffusion-weighted imaging (DWI) sequences (19). Electroencephalograms may reveal epileptiform or slow-wave activity (20). CSF analysis revealed mildly elevated protein levels and minimal leukocytosis approximately a week after symptom onset (7). Detection of HHV-6B nucleic acid in CSF aids in the diagnosis, and RT-qPCR is recommended for the detection of HHV-DNA in CSF (21, 22). Previous studies have shown that the sensitivity of NGS for detecting HHV-6 sequences is equivalent to that of real-time PCR (23). Ganciclovir and foscarnet remain the first-line agents for the treatment of HHV-6B encephalitis. The recommended dose of ganciclovir is 5 mg/kg twice daily and the recommended dose of foscarnet is 90 mg/kg twice daily (24).

Invasive Aspergillus is an important CNS OI pathogen affecting liver transplant patient prognosis, and is the leading cause of brain abscesses, with a prevalence is 0.5–0.8% (20). Usually, the abscess is located in the frontoparietal lobe, basal ganglia, cerebellum, and brainstem, with radiographic findings of single or multiple nonenhancing, low-density lesions (25). Previous studies have revealed that invasive Aspergillus infection, which usually spreads from the lungs, is usually diagnosed at a median of 25 days after OLT (26). The clinical manifestation of CNS Aspergillus infection usually presents with fever, alterations in mental status, seizures, stroke, and focal neurological deficits (27–29). Although sensitive antifungal drugs can be selected, such as voriconazole or amphotericin B, the mortality rate is high, at 65–100% (30). Unfortunately, fungal cell wall biomarkers beta-D-glucan (BDG) and galactomannan are currently not recommended for screening and diagnosis of invasive Aspergillus infection in OLT recipients because of their limited accuracy (26, 31). In particular, for CNS infections, sensitivity and specificity are low. Hong et al. suggested that NGS could be used to definitively diagnose fungal infections in the lung, bone, brain, and vascular tissues (32). Shishido reported a case of invasive Aspergillus fumigatus brain abscess definitively diagnosed using NGS after liver transplantation (33). The recipient’s serum galactomannan level was negative, and CSF PCR was negative for Aspergillus. According to several studies, voriconazole remains the recommended antifungal agent for the treatment of invasive Aspergillus, with a 50%-100% effective rate and good nervous system penetration (34–38). A meta-analysis suggested that serum concentrations between 1.0 and 6.0 mg/L during voriconazole treatment may be warranted to optimize clinical success and minimize toxicity (39). For CNS aspergillosis, the recommended trough serum concentration for treatment is at least 2 mg/L or >3 mg/L (34).

Aspergillus and Candida are the most common causes of Invasive fungal disease in liver transplantation (40). According to the guidelines from American Society of Transplantation Infectious Diseases Community of Practice, the following recommendations are made for diagnosis and medication as for common invasive Aspergillosis and Candida infections after orthotopic liver transplantation (34, 41): (1) Bronchoalveolar lavage (BAL) galactomannan (GM) and PCR can be used in combination with other fungal diagnostic modalities (e.g. chest CT-scan, culture) for the diagnosis of invasive Aspergillosis, while identification of Candida from a sterile body site by culture or visualization of the organism in tissue histopathology is the gold standard for diagnosis; (2) Voriconazole is the drug of choice to treat all forms of invasive Aspergillosis, while echinocandin and fluconazole are recommended for treatment of invasive Candida; (3) Dose of calcineurin inhibitor (CNI) and mammalian target of rapamycin (mTOR) inhibitor should be adjusted, and CNI/mTOR inhibitor levels must be monitored closely during the antifungal medication. Meanwhile, therapeutic drug monitoring (TDM) for voriconazole and posaconazole is recommended; (4) Targeted prophylaxis antifungal therapy is recommended for recipients with high risk factors such as re-transplantation, re-operation, renal failure requiring hemodialysis, according to the guidelines; (5) Anidulafungin, micafungin, caspofungin or voriconazole is recommended for the use of targeted prophylaxis against invasive Aspergillosis, and the targeted prophylaxis should be maintained for 14-21 days. As for invasive Candida, fluconazole or echinocandins is recommended as the preferred medication for targeted prophylaxis, which should be maintained for 14-28 days.

P1 was an adult male who underwent liver transplantation, whose primary disease was hepatitis B cirrhosis and hepatic cell carcinoma (HCC). It is uncommon in HHV-6 infection. HHV-6 infection is common in pediatric organ transplantation, especially in recipients younger than 3 years of age, and 35% of infected patients typically develop clinical symptoms within 2 weeks of transplantation (42). The blood concentration of tacrolimus at P1 was high before the onset of headache, with the highest concentration of 21.6 ng/mL, which appeared on POD 3. Several studies have shown that tacrolimus can induce neurotoxic symptoms ranging from mild nonspecific symptoms, such as restlessness, anxiety, tremor, headache, insomnia, severe seizures, cognitive impairment, and coma, even within the treatment range (43–45). Therefore, it needs to be distinguished from OI caused by strong immunosuppression. P2 underwent multiple plasma transfusions and artificial liver therapy before liver transplantation for ACLF. The Child–-Pugh score was 13 and the end-stage liver disease (MELD) score was 29. Previous studies have shown that MELD scores > 25, post-transplant acute kidney injury, and pre-transplant fungal colonization seem to be associated with a high risk of invasive fungal infection after liver transplantation (46, 47). Patients with decompensated cirrhosis and acute-on-chronic liver failure (ACLF), who suffer from a profound state of immune dysfunction and receive intensive care management, are susceptible to invasive fungal infection after liver transplantation (48). The duration of surgery, hours of mechanical ventilation, and preoperative dialysis days were significant factors in the development of postoperative invasive aspergillosis (49). P2’s duration of surgery was prolonged because of severe PRS during reperfusion, which induced longer mechanical ventilation and intensive care unit (ICU) duration than normal. Therefore, this situation undoubtedly increases the chances of opportunistic invasive Aspergillus infections. In addition, the successful treatment of the two patients was indispensable for the regulation of immunosuppressive agents. Effective anti-infection measures should be guaranteed, while normal graft function without rejection should be ensured. Sirolimus has been shown to have antifungal activity against several fungal pathogens, such as Candida albicans, Aspergillus, and Cryptococcus neoformans (50–52). Therefore, we believe that the antifungal effect of sirolimus is indispensable for successful treatment of P2.

Our P2 report has several limitations. A second CSF NGS examination revealed Candida spp. Owing to the low number of sequences, we believe that it may be caused by contamination of the specimen. A biopsy of the intracranial infected lesion was planned; however, P2 refused. Therefore, it is unknown whether there was a co-infection with Candida. Even if a definitive diagnosis of Candida infection was not available, the antifungal agent fluconazole was added at that time. In addition, during the later follow-up period, NGS of the CSF was not performed again, and only brain MRI was performed.

## Conclusions

In summary, our results suggest that NGS has obvious advantages for identifying pathogens and can be used as a standard test for liver transplant recipients who are suspected to suffer from OI in the CNS. In addition, anti-infection measures should not be emphasized blindly, and immunosuppressive agents should be adjusted throughout the whole treatment process.

## Ethics statement

The study was reviewed and approved by The First Affiliated Hospital of USTC Medical Research Ethics Committee in accordance with local legislative and institutional requirements. A written informed consent was obtained from the two patients for publication of the details of their medical case and accompanying image.

## Author contributions

YG designed the study, reviewed the literature, and drafted the manuscript. ZZ, WC, and ST collected and synthesized the data. DY participated in study design and critical revision of the manuscript. All authors have contributed to the manuscript and approved the submitted version.

## Acknowledgments

We thank all the medical and nursing staff involved in these two liver transplantation, postoperative management, postoperative follow-ups.

## Conflict of interest

The authors declare that the research was conducted in the absence of any commercial or financial relationships that could be construed as a potential conflict of interest.

The handling editor YM declared a shared affiliation with the authors at the time of the review.

## Publisher’s note

All claims expressed in this article are solely those of the authors and do not necessarily represent those of their affiliated organizations, or those of the publisher, the editors and the reviewers. Any product that may be evaluated in this article, or claim that may be made by its manufacturer, is not guaranteed or endorsed by the publisher.

## References

[1] Angarita SAK Russell TA Kaldas FM. Pneumonia after liver transplantation. Curr Opin Organ Transpl (2017) 22(4):328–35. doi: 10.1097/MOT.0000000000000427 28542110

[2] Heroux A Pamboukian SV. Neurologic aspects of heart transplantation. Handb Clin Neurol (2014) 121:1229–36. doi: 10.1016/B978-0-7020-4088-7.00082-1 24365414

[3] Zivković SA. Neurologic aspects of multiple organ transplantation. Handb Clin Neurol (2014) 121:1305–17. doi: 10.1016/B978-0-7020-4088-7.00089-4 24365421

[4] Pizzi M Ng L. Neurologic complications of solid organ transplantation. Neurol Clin (2017) 35(4):809–23. doi: 10.1016/j.ncl.2017.06.013 28962815

[5] Guenette A Husain S. Infectious complications following solid organ transplantation. Crit Care Clin (2019) 35(1):151–68. doi: 10.1016/j.ccc.2018.08.004 PMC7127653 30447777

[6] Fishman JA. Opportunistic infections–coming to the limits of immunosuppression? Cold Spring Harb Perspect Med (2013) 3(10):a015669. doi: 10.1101/cshperspect.a015669 24086067 PMC3784816

[7] Agnihotri SP. Central nervous system opportunistic infections. Semin Neurol (2019) 39(3):383–90. doi: 10.1055/s-0039-1687842 31378873

[8] Ablashi D Agut H Alvarez-Lafuente R Clark DA Dewhurst S DiLuca D. Classification of HHV-6A and HHV-6B as distinct viruses. Arch Virol (2014) 159(5):863–70. doi: 10.1007/s00705-013-1902-5 PMC4750402 24193951

[9] Bonnafous P Marlet J Bouvet D Salamé E Tellier AC Guyetant S. Fatal outcome after reactivation of inherited chromosomally integrated HHV-6A (iciHHV-6A) transmitted through liver transplantation. Am J Transpl (2018) 18(6):1548–51. doi: 10.1111/ajt.14657 29316259

[10] Fernández-Ruiz M Kumar D Husain S Lilly L Renner E Mazzulli T. Utility of a monitoring strategy for human herpesviruses 6 and 7 viremia after liver transplantation: a randomized clinical trial. Transplantation (2015) 99(1):106–13. doi: 10.1097/TP.0000000000000306 25073037

[11] Tang H Serada S Kawabata A Ota M Hayashi E Naka T. CD134 is a cellular receptor specific for human herpesvirus-6B entry. Proc Natl Acad Sci U S A (2013) 110(22):9096–9. doi: 10.1073/pnas.1305187110 PMC3670305 23674671

[12] Tang H Mori Y. Determinants of human CD134 essential for entry of human herpesvirus 6B. J Virol (2015) 89(19):10125–9. doi: 10.1128/JVI.01606-15 PMC4577881 26202244

[13] Agut H Bonnafous P Gautheret-Dejean A. Laboratory and clinical aspects of human herpesvirus 6 infections. Clin Microbiol Rev (2015) 28(2):313–35. doi: 10.1128/CMR.00122-14 PMC4402955 25762531

[14] Phan TL Pritchett JC Leifer C Zerr DM Koelle DM Di Luca D. HHV-6B infection, T-cell reconstitution, and graft-vs-host disease after hematopoietic stem cell transplantation. Bone Marrow Transpl (2018) 53(12):1508–17. doi: 10.1038/s41409-018-0225-2 PMC12952197 29795424

[15] Phan TL Lautenschlager I Razonable RR Munoz FM. HHV-6 in liver transplantation: A literature review. Liver Int (2018) 38(2):210–23. doi: 10.1111/liv.13506 28650593

[16] Magalhães GS Guardia AC Sampaio AM Boin IF Stucchi RS. HHV-6: clinical and laboratory investigations and correlations with encephalitis in liver transplant recipients. Transplant Proc (2013) 45(5):1997–9. doi: 10.1016/j.transproceed.2013.01.095 23769093

[17] Inui Y Yakushijin K Okamura A Tanaka Y Shinzato I Nomura T. Human herpesvirus 6 encephalitis in patients administered mycophenolate mofetil as prophylaxis for graft-versus-host disease after allogeneic hematopoietic stem cell transplantation. Transpl Infect Dis (2019) 21(1):e13024. doi: 10.1111/tid.13024 30414316

[18] Nash PJ Avery RK Tang WH Starling RC Taege AJ Yamani MH. Encephalitis owing to human herpesvirus-6 after cardiac transplant. Am J Transpl (2004) 4(7):1200–3. doi: 10.1111/j.1600-6143.2004.00459.x 15196083

[19] Seeley WW Marty FM Holmes TM Upchurch K Soiffer RJ Antin JH. Post-transplant acute limbic encephalitis: clinical features and relationship to HHV6. Neurology (2007) 69(2):156–65. doi: 10.1212/01.wnl.0000265591.10200.d7 17620548

[20] Cohen BA Stosor V. Opportunistic infections of the central nervous system in the transplant patient. Curr Neurol Neurosci Rep (2013) 13(9):376. doi: 10.1007/s11910-013-0376-x 23881624

[21] Pellett Madan R Hand J. AST infectious diseases community of practice. human herpesvirus 6, 7, and 8 in solid organ transplantation: Guidelines from the American society of transplantation infectious diseases community of practice. Clin Transpl (2019) 33(9):e13518. doi: 10.1111/ctr.13518 30844089

[22] Ogata M Uchida N Fukuda T Ikegame K Kamimura T Onizuka M. Clinical practice recommendations for the diagnosis and management of human herpesvirus-6B encephalitis after allogeneic hematopoietic stem cell transplantation: the Japan society for hematopoietic cell transplantation. Bone Marrow Transpl (2020) 55(6):1004–13. doi: 10.1038/s41409-019-0752-5 31745253

[23] Kawada J Okuno Y Torii Y Okada R Hayano S Ando S. Identification of viruses in cases of pediatric acute encephalitis and encephalopathy using next-generation sequencing. Sci Rep (2016) 6:33452. doi: 10.1038/srep33452 27625312 PMC5022051

[24] Ward KN Hill JA Hubacek P de la Camara R Crocchiolo R Einsele H. Guidelines from the 2017 European conference on infections in leukaemia for management of HHV-6 infection in patients with hematologic malignancies and after hematopoietic stem cell transplantation. Haematologica (2019) 104(11):2155–63. doi: 10.3324/haematol.2019.223073 PMC6821622 31467131

[25] Baddley JW Salzman D Pappas PG. Fungal brain abscess in transplant recipients: epidemiologic, microbiologic, and clinical features. Clin Transpl (2002) 16(6):419–24. doi: 10.1034/j.1399-0012.2002.02033.x 12437621

[26] Hogen R Dhanireddy KK. Invasive fungal infections following liver transplantation. Curr Opin Organ Transpl (2017) 22(4):356–63. doi: 10.1097/MOT.0000000000000431 28548995

[27] Kourkoumpetis TK Desalermos A Muhammed M Mylonakis E. Central nervous system aspergillosis: a series of 14 cases from a general hospital and review of 123 cases from the literature. Med (Baltimore) (2012) 91(6):328–36. doi: 10.1097/MD.0b013e318274cd77 23117848

[28] Jantunen E Volin L Salonen O Piilonen A Parkkali T Anttila VJ. Central nervous system aspergillosis in allogeneic stem cell transplant recipients. Bone Marrow Transpl (2003) 31(3):191–6. doi: 10.1038/sj.bmt.1703812 12621480

[29] Torre-Cisneros J Lopez OL Kusne S Martinez AJ Starzl TE Simmons RL. CNS aspergillosis in organ transplantation: a clinicopathological study. J Neurol Neurosurg Psychiatry (1993) 56(2):188–93. doi: 10.1136/jnnp.56.2.188 PMC1014820 8437008

[30] Potluri K Holt D Hou S. Neurologic complications in renal transplantation. Handb Clin Neurol (2014) 121:1245–55. doi: 10.1016/B978-0-7020-4088-7.00084-5 24365416

[31] Patterson TF Thompson GR 3rd Denning DW Fishman JA Hadley S Herbrecht R. Practice guidelines for the diagnosis and management of aspergillosis: 2016 update by the infectious diseases society of America. Clin Infect Dis (2016) 63(4):e1–60. doi: 10.1093/cid/ciw326 27365388 PMC4967602

[32] Hong DK Blauwkamp TA Kertesz M Bercovici S Truong C Banaei N. Liquid biopsy for infectious diseases: sequencing of cell-free plasma to detect pathogen DNA in patients with invasive fungal disease. Diagn Microbiol Infect Dis (2018) 92(3):210–3. doi: 10.1016/j.diagmicrobio.2018.06.009 30017314

[33] Shishido AA Vostal A Mayer R Ho CY Baddley JW. Diagnosis of central nervous system invasive aspergillosis in a liver transplant recipient using microbial cell-free next generation DNA sequencing. Transpl Infect Dis (2021) 23(4):e13592. doi: 10.1111/tid.13592 33655668

[34] Husain S Camargo JF. Invasive aspergillosis in solid-organ transplant recipients: Guidelines from the American society of transplantation infectious diseases community of practice. Clin Transpl (2019) 33(9):e13544. doi: 10.1111/ctr.13544 30900296

[35] Fortún J Martín-Dávila P Sánchez MA Pintado V Alvarez ME Sánchez-Sousa A. Voriconazole in the treatment of invasive mold infections in transplant recipients. Eur J Clin Microbiol Infect Dis (2003) 22(7):408–13. doi: 10.1007/s10096-003-0960-0 12827536

[36] Walsh TJ Anaissie EJ Denning DW Herbrecht R Kontoyiannis DP Marr KA. Treatment of aspergillosis: clinical practice guidelines of the infectious diseases society of America. Clin Infect Dis (2008) 46(3):327–60. doi: 10.1086/525258 18177225

[37] Singh N Husain S. AST infectious diseases community of practice. aspergillosis in solid organ transplantation. Am J Transpl (2013) 13 Suppl 4:228–41. doi: 10.1111/ajt.12115 23465016

[38] Schwartz S Reisman A Troke PF. The efficacy of voriconazole in the treatment of 192 fungal central nervous system infections: a retrospective analysis. Infection (2011) 39(3):201–10. doi: 10.1007/s15010-011-0108-6 21512792

[39] Luong ML Al-Dabbagh M Groll AH Racil Z Nannya Y Mitsani D. Utility of voriconazole therapeutic drug monitoring: a meta-analysis. J Antimicrob Chemother (2016) 71(7):1786–99. doi: 10.1093/jac/dkw099 27165788

[40] Lum L Lee A Vu M Strasser S Davis R. Epidemiology and risk factors for invasive fungal disease in liver transplant recipients in a tertiary transplant center. Transpl Infect Dis (2020) 22(6):e13361. doi: 10.1111/tid.13361 32510755

[41] Aslam S Rotstein C. AST infectious disease community of practice. candida infections in solid organ transplantation: Guidelines from the American society of transplantation infectious diseases community of practice. Clin Transpl (2019) 33(9):e13623. doi: 10.1111/ctr.13623 31155770

[42] Ylinen E Lehtinen S Jahnukainen T Karlsson T Loginov R Mannonen L. Human herpes virus 6 infection in pediatric organ transplant patients. Pediatr Transpl (2017) 21(4):e12905. doi: 10.1111/petr.12905 28213954

[43] Sato K Kobayashi Y Nakamura A Fukushima D Satomi S. Early post-transplant hyperbilirubinemia is a possible predictive factor for developing neurological complications in pediatric living donor liver transplant patients receiving tacrolimus. Pediatr Transpl (2017) 21(2):e12843. doi: 10.1111/petr.12843 27804185

[44] Umeda Y Matsuda H Sadamori H Shinoura S Yoshida R Sato D. Leukoencephalopathy syndrome after living-donor liver transplantation. Exp Clin Transpl (2011) 9(2):139–44. 21453233

[45] Xie M Rao W Sun LY Zhu ZJ Deng YL Shen ZY. Tacrolimus-related seizure after pediatric liver transplantation–a single-center experience. Pediatr Transpl (2014) 18(1):58–63. doi: 10.1111/petr.12198 24283660

[46] Raghuram A Restrepo A Safadjou S Cooley J Orloff M Hardy D. Invasive fungal infections following liver transplantation: incidence, risk factors, survival, and impact of fluconazole-resistant candida parapsilosis (2003-2007). Liver Transpl (2012) 18(9):1100–9. doi: 10.1002/lt.23467 22577087

[47] Utsumi M Umeda Y Yagi T Nagasaka T Shinoura S Yoshida R. Risk analysis for invasive fungal infection after living donor liver transplantation: Which patient needs potent prophylaxis? Dig Surg (2019) 36(1):59–66. doi: 10.1159/000486548 29649828

[48] Ferrarese A Cattelan A Cillo U Gringeri E Russo FP Germani G. Invasive fungal infection before and after liver transplantation. World J Gastroenterol (2020) 26(47):7485–96. doi: 10.3748/wjg.v26.i47.7485 PMC7754548 33384549

[49] Karadag HI Andacoglu O Papadakis M Paul A Oezcelik A Malamutmann E. Invasive fungal infections after liver transplantation: A retrospective matched controlled risk analysis. Ann Transpl (2021) 26:e930117. doi: 10.12659/AOT.930117 PMC8353998 34354035

[50] Cruz MC Goldstein AL Blankenship J Del Poeta M Perfect JR McCusker JH. Rapamycin and less immunosuppressive analogs are toxic to candida albicans and cryptococcus neoformans *via* FKBP12-dependent inhibition of TOR. Antimicrob Agents Chemother (2001) 45(11):3162–70. doi: 10.1128/AAC.45.11.3162-3170.2001 PMC90798 11600372

[51] Cruz MC Cavallo LM Görlach JM Cox G Perfect JR Cardenas ME. Rapamycin antifungal action is mediated *via* conserved complexes with FKBP12 and TOR kinase homologs in cryptococcus neoformans. Mol Cell Biol (1999) 19(6):4101–12. doi: 10.1128/MCB.19.6.4101 PMC104369 10330150

[52] Fang A Wong GK Demain AL. Enhancement of the antifungal activity of rapamycin by the coproduced elaiophylin and nigericin. J Antibiot (Tokyo) (2000) 53(2):158–62. doi: 10.7164/antibiotics.53.158 10805576

